# Pharmacological changes in cellular Ca^2+^ homeostasis parallel initiation of atrial arrhythmogenesis in murine langendorff-perfused hearts

**DOI:** 10.1111/j.1440-1681.2009.05170.x

**Published:** 2009-10

**Authors:** Yanmin Zhang, Christof Schwiening, Matthew J Killeen, Yanhui Zhang, Aiqun Ma, Ming Lei, Andrew A Grace, Christopher L-H Huang

**Affiliations:** * Physiological Laboratory, University of CambridgeCambridge; † Department of Biochemistry, University of CambridgeCambridge; ∫ Cardiovascular Research Group, School of Clinical and Laboratory Sciences, The University of ManchesterManchester, UK; ‡ Cardiovascular Research Centre, Massachusetts General Hospital and Harvard Medical School, Harvard UniversityBoston, Massachusetts, USA; § Department of Cardiovascular Medicine, First Hospital of Xi’an Jiaotong UniversityXi’an, PR China

**Keywords:** atrial arrhythmogenesis, Ca^2+^ homeostasis, murine hearts

## Abstract

1. Intracellular Ca^2+^ overload has been associated with established atrial arrhythmogenesis. The present experiments went on to correlate acute initiation of atrial arrhythmogenesis in Langendorff-perfused mouse hearts with changes in Ca^2+^ homeostasis in isolated atrial myocytes following pharmacological procedures that modified the storage or release of sarcoplasmic reticular (SR) Ca^2+^ or inhibited entry of extracellular Ca^2+^.
2. Caffeine (1mmol/L) elicited diastolic Ca^2+^ waves in regularly stimulated atrial myocytes immediately following addition. This was followed by a decline in the amplitude of the evoked transients and the disappearance of such diastolic events, suggesting partial SR Ca^2+^ depletion.
3. Cyclopiazonic acid (CPA; 0.15µmol/L) produced more gradual reductions in evoked Ca^2+^ transients and abolished diastolic Ca^2+^ events produced by the further addition of caffeine.
4. Nifedipine (0.5µmol/L) produced immediate reductions in evoked Ca^2+^ transients. Further addition of caffeine produced an immediate increase followed by a decline in the amplitude of the evoked Ca^2+^ transients, without eliciting diastolic Ca^2+^ events.
5. These findings correlated with changes in spontaneous and provoked atrial arrhythmogenecity in mouse isolated Langendorf-perfused hearts. Thus, caffeine was pro-arrhythmogenic immediately following but not >5min after application and both CPA and nifedipine pretreatment inhibited such arrhythmogenesis.
6. Together, these findings relate acute atrial arrhythmogenesis in intact hearts to diastolic Ca^2+^ events in atrial myocytes that, in turn, depend upon a finite SR Ca^2+^ store and diastolic Ca^2+^ release following Ca^2+^-induced Ca^2+^ release initiated by the entry of extracellular Ca^2+^.

## Introduction

Atrial arrhythmias constitute the most common sustained disorders of cardiac rhythm encountered in clinical practice. For example, atrial fibrillation (AF) is associated with substantial mortality and morbidity from stroke, thromboembolism, heart failure and impaired quality of life.^1^ Established atrial arrhythmogenesis has been associated with intracellular Ca^2+^ overload.^2^ Clinical and experimental studies report that atrial myocytes in situations of established AF and accompanying atrial hypertrophy,^3^ as well as in conditions such as congestive cardiac failure,^4^,^5^ show increased spontaneous diastolic Ca^2+^ release from the sarcoplasmic reticulum (SR). However, the precise cause-and-effect relationships between atrial arrhythmogenecity, any accompanying anatomical or functional remodelling and changes in SR Ca^2+^ release remain unclear. Consequently, the mechanisms involved in the initiation or termination, particularly of acute, as opposed to established, AF remain poorly understood. This acute situation is exemplified by the condition of catecholaminergic polymorphic ventricular tachycardia (CPVT), associated with cardiac ryanodine receptor (RyR2) mutations that result in episodic atrial arrhythmias, including sinus bradycardia, junctional rhythms and AF, in addition to ventricular tachyarrhythmias. These are initiated by stress and adrenergic stimulation, despite the absence of anatomical abnormalities.^6^,^7^

Rather than considering established atrial arrhythmogenesis, the present paper is concerned with the possible roles of pharmacological changes to cellular Ca^2+^ homeostasis in the acute initiation of atrial arrhythmogenecity in otherwise functionally and structurally normal hearts. Previous studies on atrial systems have not made such correlations.^8^–^10^ Mouse rather than canine,^4^ rabbit^11^ or rat hearts^9^ were used in view of their potential importance in providing genetic arrhythmic models, as has been the case for ventricular arrhythmogenesis in LQTS3,^12^ Brugada syndrome^13^ and CPVT.^14^–^16^ Rather than using pulmonary vein preparations, which also include smooth and pacemaker-like cells, we studied isolated atrial myocytes, which include smooth and pacemaker-like cells in addition to atrial myocyte-like cells.^17^ Thus, our approach required modifications in both atrial myocyte isolation procedures to improve cell viability and yields for confocal Ca^2+^ imaging and a separation of atrial from ventricular electrophysiological activity in intact Langendorf-perfused mouse hearts through a range of pacing conditions.

## Methods

Inbred 129/Sv wild-type mice (Harlan, Bicester, UK) were kept in an animal house at room temperature under a 12h light–dark cycle and fed sterile rodent chow with constant access to water. All procedures conformed to the UK Animals (Scientific Procedures) Act (1986).

### Solutions

The following solutions were used during the course of the preparation and experimental procedures. Solution A for electrophysiological experiments on Langendorff-perfused hearts consisted of normal bicarbonate-buffered Krebs’–Henseleit (KH) solution (composition (in mmol/L): NaCl 119; NaHCO_3_ 25; KCl 4.0; KH_2_PO_4_ 1.2; MgCl_2_ 1.0; CaCl_2_ 1.8; glucose 10; sodium pyruvate 2.0) maintained at pH7.4 by bubbling with 95% O_2_–5% CO_2_ (British Oxygen, Manchester, UK). Solution B was the basic solution from which other solutions used in the myocyte isolation procedure were derived (composition (in mmol/L): NaCl 125; KCl 4.75; MgSO_4_ 1.2; KH_2_PO_4_ 1.2; HEPES 30; glucose 10; taurine 50, titrated to pH7.4 with NaOH). The solution was filtered through a 0.2µm filter (Millipore, Billerica, MA, USA) to remove microbes and small particles. This provided the stock solution for the production of Solutions C–H used during myocyte isolation.

1. Solution C: this 750µmol/L Ca^2+^-containing perfusion solution was prepared by adding 750µmol/L CaCl_2_ to Solution B.
2. Solution D: this Ca^2+^-free nitrilotriacetic acid (NTA)-containing solution was prepared by adding 5mmol/L NTA to Solution B and titrating the resulting solution to pH6.95 with NaOH.
3. Solution E: an enzyme solution prepared by adding 1.5mg/mL collagenase (Worthington Type II), 2mg/mL hyaluronidase (Sigma, Gillingham, Dorset, UK) and 100µmol/L CaCl_2_ to Solution B.
4. Solution F: this was a further digestion buffer made by adding 1mg/mL collagenase and 1mg/mL bovine servum albumin (BSA) to Solution B.
5. Solution G: this was an enzyme washout solution made by adding 1mg/mL BSA and 250µmol/L CaCl_2_ to Solution B.
6. Solution H: this Ca^2+^-containing solution was made by adding 1.2mmol/L CaCl_2_ to Solution B.

After preparation, Solutions D–G were always filtered using a 0.2µm filter to remove microbes and small particles.

### Atrial myocyte isolation

Atrial myocytes were obtained from mice aged 3 months. Single mouse atrial myocytes used for the imaging of Ca^2+^ signals were isolated using an enzymatic digestion protocol substantially modified and combined from previously established procedures.^18^,^19^ Mice were killed by cervical dislocation (Schedule I, UK Animals (Scientific Procedures) Act, 1986). Hearts were then rapidly excised and cannulated in ice-cold Krebs’–Henseleit solution (Solution A) before mounting onto a Langendorff perfusion system for perfusion with Solution C (for 4min), Solution D (4min) and Solution E (10–12min) in sequence at a stable temperature of 37°C. The heart was then removed from the perfusion apparatus and the atrial appendages were excised and chopped into several pieces in Solution F. These were further incubated for another 5–10min with gentle manual agitation using a 1mL tip transfer pipette. All these latter steps were performed at 36–37°C. Cells were then separated from the enzymatic solution by centrifuging at 243*g* for 3min. The resulting isolated cells were then washed using Solution G, followed after 5min by centrifugation at 30*g* for 2min. The cells were then resuspended in Solution H and, after a 5min interval, centrifuged again at 30*g* for 2min. The cells were then maintained at room temperature in Solution H for the experiments that followed, in common with previous studies in atrial myocytes.^3^,^20^–^22^ We observed that cardiac myocytes were more viable studied under these conditions than at the higher temperatures used when perfusing whole hearts. Accordingly, our studies sought to investigate the presence or absence of particular effects, rather than making full quantitative comparisons between single myocytes and whole hearts.

### Confocal microscopy

Cells were then placed on a Grade 1 circular laminin-coated coverslip (Menzel, Glasbearbeitungswerk, Germany) that formed the floor of a 1.5mL perfusion chamber, to which it was fixed with vacuum grease. Cells were then loaded with the acetoxymethyl (AM) ester of Fluo-3 (Molecular Probes, Leiden, The Netherlands) by incubation with 5µmol/L Fluo-3 AM in Solution H (1.2mmol/L CaCl_2_) for 10–20min in the dark before washout of the Fluo-3-containing solution. Cells were then transferred onto the stage of a Zeiss LSM-510 laser scanning confocal system (Zeiss, Jena, Germany) with a ×20 air objective on a Zeiss Axiovert 100M inverted microscope. Fluo-3 fluorescence emission was excited with a 488nm argon laser and measured at wavelengths between 505 and 550nm. Images were then analysed using an in-house custom-made software program. Series of 500 frames (128×64pixels/frame) were collected at a scanning frequency of 25msec/frame to monitor fluorescence changes over time. Fluorescence measurements, corrected for background signal in regions outside the cells, were made within defined regions of interest (F) and were normalized to their resting fluorescence (F_0_) values. For each of the myocytes studied, peak F/F_0_ values were calculated throughout each time series acquired and a mean peak F/F_0_ was calculated for that series. Where indicated, cells were paced at 1Hz (5V above excitation threshold of 30–60V for 2msec) with two field electrodes. All fluorescence studies were performed at room temperature. Ca^2+^ transients were measured both from regions of interest (ROIs) covering entire cells and from sets of three (1×4 pixel; 1 pixel=0.6×0.6µm) ROIs placed at the cell margin, the cell centre and regions between these, approximately 2–3µm from the surface.

### Langendorff preparations for atrial electrophysiological experiments

The whole-heart experiments used modifications of previously established procedures to set up a Langendorff perfusion system for the murine heart.^23^–^25^ Male and female mice (age 3–6 months) were randomly selected and injected with 50IU, i.p., heparin 10–15min before being killed by cervical dislocation (Schedule I, UK Animals (Scientific Procedures) Act, 1986). We observed that hearts from younger mice were significantly less amenable to successful cannulation. The heart was cannulated *in situ* using a straight-cut and smoothed 21gauge needle previously filled with Solution A, dissected and then fixed securely with a straight 60g pressure microaneurysm clip (Harvard Apparatus, Edenbridge, UK). The cannulated heart was perfused with Solution A at room temperature before being mounted onto a Langendorff system^25^ and then perfused at a constant flow rate of 2–2.5mL/min (model 505S; Watson-Marlow Bredel Peristaltic Pumps, Falmouth, Cornwall, UK) with Solution A.

The perfusate was first filtered through 200 and 5µm membranes (Millipore UK, Watford, UK) and warmed to 37°C by a water-jacketed heat-exchange coil (Model C-58A; Techne, Cambridge, UK) before entering the coronary arterial network. The aortic valve was shut by the pressure of the perfusate that ultimately drained through the vena cava. Viable hearts regained a pink appearance and spontaneous rhythmic contractions upon warming. Hearts were perfused retrogradely for not less than 10min in the absence of stimulation. Experiments were only performed in intact Langendorff preparations showing clearcut 1:1 atrioventricular (AV) conduction during the intrinsic activity following cannulation. The *in situ* cannulation procedure gave preparations with higher intrinsic rates corresponding to baseline cycle lengths of 155.0±58.4msec (*n*=33 hearts) and more consistent 1:1 AV conduction (PR intervals of 31.8±5.3msec; *n*=33 hearts) than hearts cannulated following prior separation and immersion in ice-cold buffer (220.1±71.6msec; *n*=20 hearts).

### Electrophysiological experiments

The electrophysiological studies performed in isolated perfused hearts were designed to distinguish atrial from ventricular bipolar electrogram (BEG) waveforms and involved comparisons of records from simultaneous recordings made at two sites. Thus, in addition to the paired platinum stimulating electrodes placed on the right atrium, two bipolar recording electrodes of 1mm interpole spacing were placed on the left atrium and left ventricle. At the beginning of each experiment, the ventricular recording electrode was placed at a series of positions at successively greater distances from the atria until there was no demonstrable atrial far-field deflection in the ventricular traces while ensuring persistent far-field deflections in the atrial traces. Hearts were initially paced for not less than 5min at 10Hz to permit them to regain their physiological steady state. Three types of pacing protocols were used: (i) hearts were studied at their intrinsic rates in the absence of stimulation; (ii) hearts were subject to regular pacing at 10Hz using 2msec square-wave stimuli set at 2× the excitation threshold (Grass S48 stimulator; Grass-Telefactor, Slough, UK); and (iii) hearts were studied using a programmed electrical stimulation (PES) procedure adopted from clinical techniques used previously in ventricular studies^25^,^26^ but recently introduced in clinical studies of atrial electrophysiology.^27^ These began using standard baseline pacing stimuli at frequencies of 10Hz for 20s. Drive trains of eight paced beats (S1) were each followed by an extra stimulus (S2) every ninth beat, initially at an S1–S2 interval equal to the pacing interval. Each subsequent cycle reduced the S1–S2 interval by 1msec until atrial refractoriness was reached. The resulting electrogram signals were amplified, band-pass filtered (30Hz to 1kHz; Gould 2400S; Gould-Nicolet Technologies, Ilford, Essex, UK) and digitized at a sampling frequency of 5kHz (CED1401*plus*; Cambridge Electronic Design, Cambridge, UK).

The present protocols differed from previous studies on ventricular arrhythmogenesis^12^,^13^,^25^ in requiring hearts to be paced from the atria rather than the ventricles. A pacing rate of 8Hz permitted atrial escape phenomena that precluded regular activation by the stimulus train. This was particularly the case in studies performed in the presence of caffeine. This necessitated a higher pacing rate of 10Hz to ensure regular atrial stimulation under all the pharmacological conditions tested. However, in some hearts, such higher pacing rates resulted in a gradual development of an AV block. Nevertheless, withdrawal of the regular pacing allowing a resumption of intrinsic activity, permitted restoration of normal 1:1 AV conduction once pharmacological agents were withdrawn. These findings likely relate to refractoriness in the AV node at high pacing rates. Thus, direct measurements showed that the atria had shorter refractory periods (24±7msec; *n*=33) than the AV node (61±12msec; *n*=5).

### Statistical analysis

Statistical analysis was performed using a repeated-measures one-way anova to compare data using spsssoftware (SPSS, Chicago, IL, USA). Results from individual hearts acquired during pharmacological intervention were compared with their respective untreated controls using one-way anova for correlated samples (spsssoftware). *P*<0.05 was considered significant. Cross-tabulations with Chi-squared or Fisher's exact test were used as appropriate for categorical variables.

Data are expressed as the mean ± SEM. For experiments with single cells, *n* denotes the number of peaks from which F/F_0_ values were obtained; the numbers of cells involved are given separately. For experiments in whole hearts, *n* denotes the number of whole hearts studied.

### Agents

All drugs and other chemical agents were purchased from Sigma-Aldrich (Poole, UK), unless indicated otherwise. Nifedipine was dissolved in 96% ethanol to make a 1mmol/L stock solution, kept wrapped in foil to prevent light degradation and was kept refrigerated at 4°C. Cyclopiazonic acid (CPA) was prepared in 96% ethanol to make a 10mmol/L stock solution and was stored at –20°C. Final drug concentrations were achieved by dilution with Solution A for electrophysiological experiments and Solution H for experiments in single cells. Caffeine was dissolved directly in Solution A or H, as appropriate, and kept at room temperature.

## Results

### Ca^2+^ transients in resting atrial myocytes

**Fig. 1:**
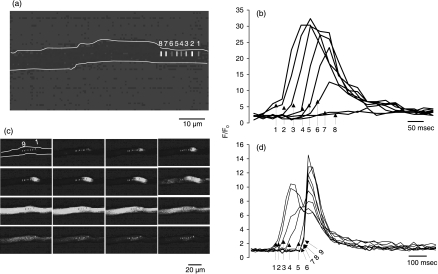
Spontaneous Ca^2+^ transients in murine atrial myocytes under resting conditions. (a) Localized 4×1 pixel regions of interest (ROIs) spaced at intervals of 2–3µm along the cell length (1–8). (b) F/F_0_ records reflecting Ca^2+^ wave propagation through ROIs (1–8). (c) Series of frames demonstrating initiation and spread of a Ca^2+^ wave. The first frame shows positions of the localized 4×1 pixel ROIs (1–9) placed at fixed positions, spaced at intervals of 2–3µm, along the cell length. (d) Corresponding F/F_0_ records from ROIs 1–9.

The murine atrial myocytes obtained using our modified procedure were viable for up to 6–8h, appearing elongated with rounded but tapered ends (length 90.4±35.6µm (range 59.5–167.4µm); width 13.1±2.0µm (range 10.1–17.2µm); *n*=15 cells) with well-defined striations. We studied a total of 90 cells from seven hearts. Of these cells, we examined the resting unstimulated properties in approximately 20 cells. The remaining cells were subjected to stimulation protocols in the absence of (*n*=15 cells) or following the addition of caffeine (*n*=16 cells), CPA (*n*=10 cells), nifedipine (*n*=8 cells), CPA+ caffeine (*n*=14 cells) or nifedipine+caffeine (*n*=5 cells). Even the approximately 20 resting, unstimulated, Fluo-3 loaded cells exhibited two forms of spontaneous Ca^2+^ activity, with some cells showing evidence of both (neither previously reported in normal ventricular cells^28^,^29^). First, approximately six of 10 cells showed spontaneous periodic Ca^2+^ waves, associated in earlier studies with a propagated Ca^2+^-induced Ca^2+^ release (CICR). This was demonstrated by comparing signals from regularly spaced ROIs (4×1 pixels; 1 pixel= 0.6×0.6µm) positioned at 2–3µm intervals along the cell lengths (ROIs designated from 1 to 8 and labelled as such in Fig.1a correspond to arrows marked 1–8 in Fig.1b). These propagated along part or all of the cell length (Fig.1c), at a relatively constant approximate 89µm/s, a velocity sufficient to account for previously reported cellular activation delays attributable to a centripetal propagation of cell activation.^21^ These waves either declined in amplitude with distance (Fig.1b) or culminated in a second type of larger synchronized event involving the entire cell, resulting in peak F/F_0_ values as high as approximately 14 (Fig.1d; ROIs labelled 1–9).

### Early and delayed effects of caffeine on Ca^2+^ transients in stimulated atrial myocytes

**Fig. 2:**
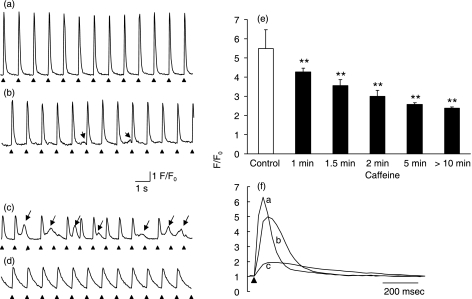
Effects of caffeine on Ca^2+^ transients from regularly stimulated atrial myocytes. (a–d) Ca^2+^ transients obtained in a typical control myocyte (a) and cells 2 (b), 5 (c) and 13min (d) after the addition of 1.0mmol/L caffeine, showing diastolic transients (arrows; b,c) and the prolonged development of a decline in signal amplitude and a prolongation of time-course (d). (e) Peak F/F_0_ values (mean±SEM) under control conditions compared with corresponding values at successively greater intervals (1, 1.5, 2, 5, >10min) after the addition of 1.0mmol/L caffeine. **P<0.01 compared with control. (f) Comparison of the timecourses of typical Ca^2+^ transients under control conditions (trace a) and <5min (trace b) and >10min (trace c) after the addition of 1.0mmol/L caffeine.

The following experiments studied Ca^2+^ transients in regularly stimulated (1Hz) Fluo-3-loaded atrial myocytes exposed to agents that sought to increase the release of SR Ca^2+^ through CICR or to inhibit SR uptake of cytosolic Ca^2+^ or extracellular Ca^2+^ entry. In each cell, Ca^2+^ transients were measured from both ROIs covering entire cells and sets of three (1×4 pixel; 1 pixel=0.6×0.6µm) ROIs placed at the cell margin, the cell centre and regions between these, approximately 2–3µm from the surface. Before the addition of pharmacological agents, myocytes showed regular successions of entrained Ca^2+^ transients with stable amplitudes of mean F/F_0_ 5.49±0.97 (from *n*=176 peaks), determined from ROIs covering the entire cell area, that decayed to a stable baseline (Fig.2a,e). These Ca^2+^ transients would be the result of cycles of depolarization-induced SR Ca^2+^ release into the cytosol and its subsequent return from the cytosol to stores whose magnitudes and time-courses would be sensitive to manoeuvres affecting either process. The addition of caffeine at a concentration identical to that used in previous studies in ventricular myoctyes^28^,^29^ initially resulted in the immediate appearance of diastolic Ca^2+^ transients that were smaller in amplitude but often more prolonged than the evoked transients (Fig.2b,c). These followed 50 of 200 peaks recorded at <5min following the introduction of caffeine in all 16 regularly stimulated cells studied (Table1). The peaks appeared to increase in amplitude from the time of application to approximately 5min, consistent with enhanced Ca^2+^ release produced by caffeine action on RyR2 Ca^2+^ release channels. This would increase cytosolic Ca^2+^, which, in turn, would also enhance CICR. However, such diastolic Ca^2+^ release would eventually deplete SR Ca^2+^ stores. The latter would be consistent with the subsequent decline in amplitude and frequency and final disappearance of the diastolic peaks in the succeeding 5–15min (Fig.2d). There was also a progressive decline in peak F/F_0_ of the evoked transients to 4.28±0.20, 3.56±0.32, 2.99±0.32, 2.57±0.08 and 2.37±0.08 at 1, 1.5, 2, 5 and 10min after caffeine addition, respectively (*n*=84, 53, 43, 36 and 88 peaks, respectively), consistent with a partial depletion of SR Ca^2+^ stores (Fig.2e). There was a corresponding prolongation of their time-courses evident from comparisons of control traces and records obtained <5min and >10min after the addition of caffeine (Fig.2f, traces a–c, respectively) that was compatible with changes in the period during which there was a net release of SR Ca^2+^. Thus, caffeine progressively lengthened the full-width half-maxima (FWHM) from 119±8 to 126±16 and 302±42msec <5min and >10min after its addition, respectively (*P<*0.01; Table2). All such FWHM readings described here were obtained from a total of 12 peaks obtained from four cells.

**Table 2:** Full-width half-maxima values for Ca^2+^ transients obtained from regularly stimulated atrial myocytes under different conditions

Agents	No. peaks	FWHM (msec)
Control	12	119±8
Caffeine <5min	12	126±16
Caffeine >5min	12	302±42*
CPA <5min	12	102±17
CPA >15min	12	128±11
CPA+caffeine	12	170±13*
Nifedipine	12	114±19
Nifedipine+caffeine	12	173±24*

Data are the mean±SEM of results based on 12 peaks obtained from four cells in each experimental group.

* *P<*0.01 compared with control.

FWHM, full-width half-maxima. CPA, cyclopiazonic acid.

**Table 1:** Occurrence of diastolic calcium release in atrial myocytes

Agent	No. cells showing diastolic release/total no. cells	No. peaks	Diastolic release	
			<5min	>5min
Control	0/15	176	0	0
Caffeine	16/16‡	200	50*†	0
CPA	3/10§	130	9*	0
Nifedipine	0/8§	96	0	0
CPA+caffeine	0/14§	179	0	0
Nifedipine+caffeine	0/5§	60	0	0

* *P*<0.01 compared with control (Chi-squared test);

† *P*<0.01 compared with cyclopiazonic acid (CPA; Chi-squared test);

‡ *P*<0.01 compared with control and CPA (Fischer's exact test);

§ *P*>0.05 compared with control (Fischer's exact test).

### Early and delayed effects of CPA on Ca^2+^ transients

**Fig. 3:**
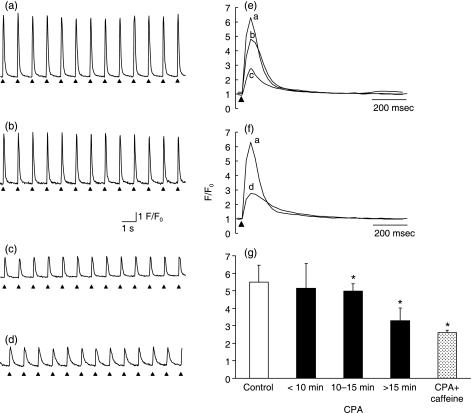
Effects of cyclopiazonic acid (CPA) on Ca^2+^ transients from regularly stimulated atrial myocytes. (a–d) Control records (a) compared with results 2 (b) and 11min (c) after the addition of 0.15µmol/L CPA and the subsequent addition of 1.0mmol/L caffeine (d), showing delayed decreases in signal amplitude (c) that persist after the addition of caffeine (d), but an absence of diastolic Ca^2+^ transients. (e,f) Comparison of the time-course of Ca^2+^ transients obtained under control conditions (trace a) and <5min (trace b) and >15min (trace c) after the addition of CPA, as well as after the addition of 1.0mmol/L caffeine following CPA pretreatment (trace d). (g) Peak F/F_0_ values (mean±SEM) under control conditions compared with corresponding values at successively greater intervals (<10, 10–15 and >15min) after the addition of 0.15µmol/L CPA and the further addition of 1.0mmol/L caffeine. *P<0.01 compared with control.

The simplest hypothesis describing the above findings would suggest that caffeine increases the tendency for SR RyR2 Ca^2+^ release channels to release Ca^2+^. However, this would then tend to deplete SR Ca^2+^ content. This would reduce levels of releasable SR Ca^2+^. The latter would, in turn, explain the observed declines in both the peak amplitude of evoked Ca^2+^ transients and the frequency of the diastolic Ca^2+^ transients following longer (>10min) exposures to caffeine. Such a hypothesis was corroborated by the results of independent pharmacological changes in such SR Ca^2+^ stores and extracellular Ca^2+^ entry using CPA and nifedipine, respectively. Following the addition of CPA, atrial cells showed only nine diastolic Ca^2+^ transients over 130 evoked peaks and then in only three of 10 cells at <5min following the addition of CPA (Fig.3a–c). Reductions in Ca^2+^ signal amplitude occurred only at >10min to peak F/F_0_ of 5.14±1.42 (*n*=78 peaks) at 10–15min and 3.29±0.73 (*n*=39 peaks) at >15min, respectively (Fig.3c,g), consistent with the more gradual reduction of SR Ca^2+^ expected from inhibition of Ca^2+^-ATPase mediated Ca^2+^ reuptake. The FWHM values remained unchanged (102±17msec at 5min and 128±11msec at 15min;*P*>0.05; Fig.3e), confirming expectations that CPA should not affect Ca^2+^ release kinetics. Further addition of caffeine now produced neither diastolic Ca^2+^ peaks nor further changes in peak F/F_0_: the peak F/F_0_ values in cells treated with CPA+caffeine, caffeine alone (>10min) and CPA alone (>15min) were statistically similar at 2.61±0.13 (*n*=52 peaks), 2.37±0.08 (*n*=88 peaks) and 3.29±0.73 (*n*=39 peaks), respectively (Fig.3g), but FWHM was prolonged to 170±13msec (Fig.3f) relative to either untreated controls (Fig.3e,f,trace a in each) or CPA-pretreated cells (*P<*0.01), consistent with effects of caffeine in prolonging Ca^2+^ release (Table2).

### Effects of nifedipine on Ca^2+^ transients

**Fig. 4:**
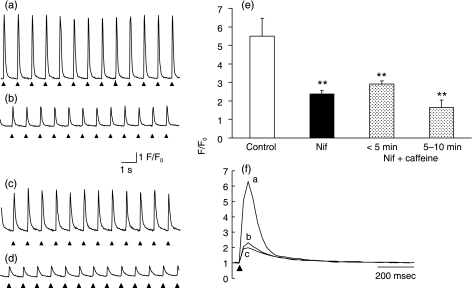
Effects of nifedipine on Ca^2+^ transients from regularly stimulated atrial myocytes. (a–d) Ca^2+^ transients in a typical control myocyte (a), in a 0.5µmol/L nifedipine-treated atrial myocyte (b) and in myocytes 2 (c) and 10min (d) after the further addition of 1.0mmol/L caffeine. (e) Peak F/F_0_ values (mean±SEM) under control conditions compared with corresponding values in the presence of nifedipine (Nif) and <5min and 5–10min after the further addition of caffeine. **P<0.01 compared with control. (f) Comparisons of the time-courses of Ca^2+^ transients obtained under control conditions (trace a) and following addition of nifedipine (0.5µmol/L) before (trace b) and after (trace c) the further addition of caffeine.

Experiments that examined the dependence of the diastolic Ca^2+^ transients induced by caffeine on extracellular Ca^2+^ entry demonstrated that nifedipine produced a prompt decrease in peak F/F_0_ (to 2.38±0.19; *n*=96 peaks; Fig.4a,b), with no changes in FWHM (114±19msec; *P*>0.05; Fig.4f, trace b) relative to control traces (Fig.4f, trace a), suggesting that it left Ca^2+^ release kinetics intact. Although further addition of caffeine increased peak F/F_0_ amplitudes to 2.90±0.39 (*n*=60 peaks), it did not result in the appearance of diastolic Ca^2+^ transients (Fig.4c,d), but did produce an accentuated decline in peak F/F_0_ (to 1.65±0.26; *n*=36 peaks; Fig.4e) and a prolongation of FWHM to 122±18msec (*P*>0.05) at approximately 5min and to 173±24msec (*P<*0.01) at 10min (Fig.4f).

### Spatial variations in Ca^2+^ signalling during pharmacological treatment

**Fig. 5:**
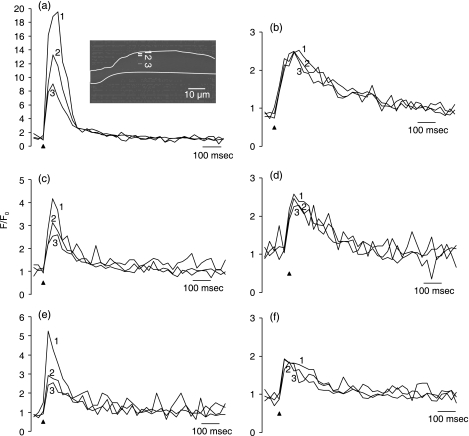
Spatial heterogeneities in evoked atrial Ca^2+^ transients. Spatial heterogeneities were analysed by three 4×1 pixel regions of interest (ROIs; 1, 2 and 3) placed at successively increasing distances from the cell margin to the cell centre at approximately 2–3µm intervals. (a) Typical results from a control atrial myocyte showing significant F/F_0_ spatial heterogeneities, as reflected in the corresponding traces (1–3), with the highest peak F/F_0_ at the cell margin and the lowest F/F_0_at the cell centre. (b) Ca^2+^ transients obtained 13min after the addition of 1.0mmol/L caffeine. (c,d) Ca^2+^ transients obtained 20min after the addition of 0.15µmol/L CPA before (c) and after (d) the addition of 1.0mmol/L caffeine. (e,f) Ca^2+^ transients obtained in the presence of 0.5µmol/L nifedipine before (e) and after (f) the addition of 1.0mmol/L caffeine.

Previous studies have implicated major participation of a propagated, centripetal CICR initiated by surface Ca^2+^ entry in atrial excitation–contraction coupling, which results in spatial gradients in Ca^2+^ signalling with distance from the cell surface.^21^ Changes in such gradients provide a useful indication of the Ca^2+^ sensitivity of the CICR process responsible for atrial activation. Therefore, in the present study, Ca^2+^ transients were measured from sets of three (1×4 pixel; 1 pixel=0.6×0.6µm) ROIs placed at the cell margin, the cell centre and regions between these, approximately 2–3µm from the surface. The present findings are compatible with an effect of caffeine in changing the characteristics of such CICR; such a hypothesis would predict that caffeine, but not nifedipine or CPA, would enhance its propagation with distance from the cell surface. Figure5 exemplifies such predictions. Under control conditions (Fig.5a), the amplitudes of evoked Ca^2+^ transients obtained from localized 4×1 pixel (2.4× 0.6µm**)**ROIs (Fig.5a, inset 1, 2 and 3) progressively decreased with distance from the cell margin, confirming previous reports.^21^,^30^–^32^ These heterogeneities persisted in the presence of CPA (Fig.5c) and nifedipine (Fig.5e). However, they were abolished by caffeine whether applied alone (Fig.5b) or in combination with either CPA or nifedipine (Fig.5d,f), consistent with an action of this agent on a centripetal propagation of Ca^2+^ release through a CICR-dependent mechanism.

### Correlations with arrhythmogenic tendency in intact hearts

The changes in cellular Ca^2+^ homeostasis correlated closely with atrial arrhythmogenic tendency in intact Langendorff-perfused hearts. Control experiments (*n*=34 hearts) assessed whether atrial arrhythmogenesis was initiated during 10–15min of intrinsic activity, 5min of regular pacing at 10Hz and following application of the PES protocol, with stimulation applied at the right atrium. These control experiments recorded episodes of either atrial tachycardia (AT), in the form of a normal sequence of electrical waveforms at increased frequency, or AF, in the form of irregularly irregular electrical deflections, extending for more than five deflections during intrinsic or regular pacing and for >1s following PES. Similar protocols were applied in experiments that then involved 20min pretreatment with CPA or nifedipine. Experiments that investigated the effect of caffeine used PES and regular pacing both immediately after and >5min after application; this was in parallel with observations in isolated myocytes in which diastolic Ca^2+^ events were observed immediately following, but not 5min after, the addition of caffeine.

**Fig. 6:**
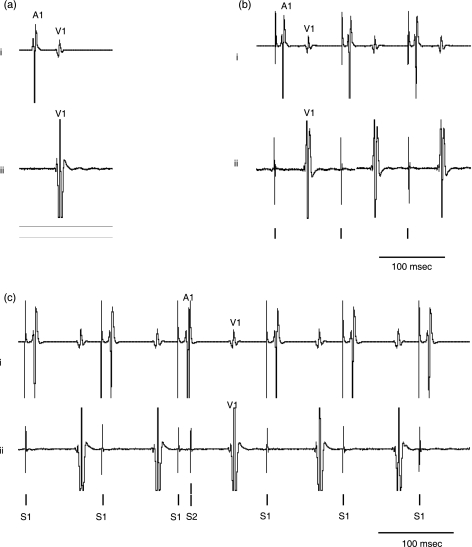
Resolution of atrial and ventricular electrogram components obtained by simultaneous recordings from two pairs of recording electrodes placed on the left atrium and left ventricle, respectively, in control hearts. (a) Results obtained during intrinsic activity showing the simultaneously obtained atrial trace (i) and ventricular trace (i), in which A1 exemplifies atrial and V1 exemplifies ventricular contributions to the respective electrogram waveforms. (b) Results obtained during regular atrial pacing at 10Hz. The atrial trace (i) shows large atrial deflections (A1) following the stimulus artefacts, followed by smaller ventricular deflections (V1). (c) Results obtained during programmed electrical stimulation (PES). Traces i and ii illustrate atrial (A1) and ventricular waveforms (V1) following each S1 stimulus and include results from an S2 stimulus imposed at an interval close to the atrial refractory period showing neither atrial nor ventricular deflections following the S2 stimulus despite normal atrial (A1) and ventricular (V1) deflections in response to the preceding S1 stimulus.

**Fig. 7:**
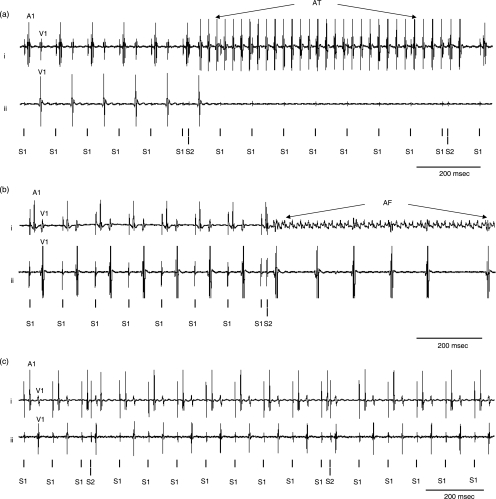
Atrial arrhythmogenic effects of caffeine. (a,b) Traces obtained <5min after the addition of 1.0mmol/L caffeine during programmed electrical stimulation (PES) showing (a) atrial tachycardia (AT; trace i) and (b) atrial fibrillation (AF; trace i) not apparent in the corresponding ventricular traces (trace ii). (c) In contrast, arrhythmogenesis was not induced in either atrial (trace i) or ventricular (trace ii) traces >5min after the addition of 1.0mmol/L caffeine.

**Fig. 8:**
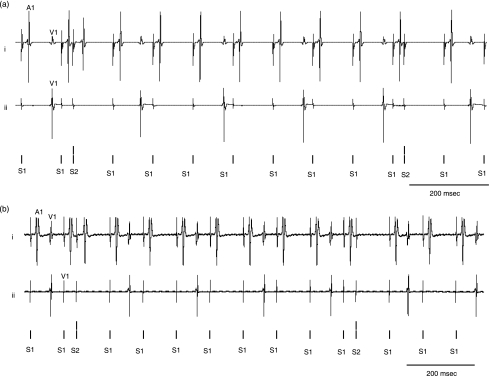
Cyclopiazonic acid (CPA) and nifedipine pretreatment abolish the caffeine-induced arrhythmogenic effect. Atrial (i) and ventricular (ii) electrograms obtained in experiments with 0.15µmol/L CPA (a) or 0.5µmol/L nifedipine (b) pretreatment prior to the addition of 1mmol/L caffeine during programmed electrical stimulation. A1, atrial waveform; V1, ventricular waveform.

Figure6 shows results obtained in control hearts, during intrinsic activity (Fig.6a), regular pacing (Fig.6b) and at the end of a typical PES procedure in which the S2 stimulus was imposed at an S1–S2 interval close to the atrial refractory period (Fig.6c). Bipolar electrogram recordings were obtained not only from the left atria, but also from the left ventricles. This simultaneous recording made it possible to distinguish the atrial deflections from ventricular far-field artefacts in the atrial records (e.g. Fig.6b, trace i) Thus, activity in the atria, did not produce far-field deflections in the corresponding ventricular traces owing to the greater distance of the ventricular electrodes from the atria (Fig.6b, trace ii). Conversely, the site of the atrial recording electrodes was comparatively close to the ventricle and therefore did record its far-field deflections. Consequently, atrial BEG recordings showed both atrial and ventricular deflections (Figs6–8, all traces marked i), but atrial deflections were absent from the ventricular BEG traces (Figs6–8, all traces marked ii). This made it possible to separate atrial from ventricular activity and thereby identify atrial as opposed to ventricular arrhythmogenesis. Such a comparison demonstrated an absence of either atrial or ventricular arrhythmogenesis through all the stimulation protocols explored. In contrast, episodes of AT (Fig.7a) or AF (Fig.7b) were observed during PES immediately following caffeine administration (*n*=11 hearts), but not after >5min exposure to caffeine (*n*=11 hearts; Fig.7c) or after CPA (*n*=11 hearts; Fig.8a) or nifedipine pretreatment (*n*=12 hearts; Fig.8b). Table3 quantifies these findings, demonstrating significant (*P<*0.05, Fisher's exact test) increases in the incidence of atrial arrhythmias only in the case immediately following the addition of caffeine and not with prolonged caffeine exposure or manoeuvres involving CPA (*n*=6 hearts) or nifedipine (*n*=6 hearts) pretreatment. These results in intact hearts precisely parallel the occurrence of diastolic Ca^2+^ transients in isolated atrial myocytes.

**Table 3:** Occurrence of arrhythmogenesis (atrial tachycardia or atrial fibrillation) under different protocols

Agents	Intrinsic (*n*)	Regular pacing at 10Hz (*n*)	PES (*n*)
Control	0 (34)	0 (34)	4 (34)
Caffeine <5min	2 (11)	3 (11)*	5 (11)*
Caffeine >5min	0 (11)	0 (11)	0 (11)
CPA pre-treated	0 (11)	0 (11)	1 (11)
CPA+caffeine	0 (6)	0 (6)	0 (6)
Nifedipine pretreated	0 (12)	0 (12)	3 (12)
Nifedipine+caffeine	0 (6)	1 (6)	0 (6)

* *P<*0.01 compared with control.

PES, programmed electrical stimulation; CPA, cyclopiazonic acid.

## Discussion

The present study investigated the possible roles of altered Ca^2+^ homeostasis in the acute initiation of atrial arrhythmogenesis in mouse intact hearts. The findings demonstrated that such arrhythmogenic phenomena depend on both a definite SR Ca^2+^ store and the diastolic release of Ca^2+^. Accordingly, atrial arrhythmogenecity was increased by pharmacological manipulations that increased SR Ca^2+^ release and this effect was abolished by either inhibiting this release or depleting SR Ca^2+^. Thus, in atrial myocytes, caffeine produced diastolic Ca^2+^ events immediately following, but not >5min after, its application, consistent with an initially increased CICR subsequently resulting in the partial depletion of a finite SR Ca^2+^ store. Both CPA and nifedipine pretreatment abolished these effects. In whole hearts, caffeine correspondingly produced pro-arrhythmogenic effects immediately following, but not >5min after, application that were abolished by CPA or nifedipine pretreatment.

Atrial myocytes show important differences from ventricular myocytes, particularly in their tubular and SR membrane systems, which may reflect functional differences in their Ca^2+^ homeostatic processes. Atrial myocytes do not possess extensive T-tubular systems^8^,^21^ and, instead, have prominent transversely orientated SR, Z-tubular, elements. Atrial cells show an abundant corbular SR containing non-junctional RyR2.^33^ Junctional RyR2–L-type Ca^2+^ channel (LTCC) clusters are confined to the cell peripheries.^8^ This may reflect atrial activation normally involving CICR in a pattern of centripetal propagation into the cell interior from superficial T–SR junctions.^8^,^21^,^30^ We then examined the acute effects of caffeine on isolated atrial myocytes. Caffeine is thought to increase the release of intracellularly stored Ca^2+^, either by sensitizing RyR2 to cytosolic Ca^2+^ or inhibiting phosphodiesterase activity, thereby increasing cellular cAMP^34^ and consequently increasing the open probabilities of the RyR2-channels.^35^ Therefore, caffeine would be expected to initially result in abnormal RyR2-mediated diastolic SR Ca^2+^ release that would subsequently cease with the resulting reduction in SR Ca^2+^, as reported previously for ventricular cells.^36^,^37^

In regularly stimulated single murine atrial myocytes, application of caffeine resulted in the early appearance of diastolic Ca^2+^ release. However, there was a subsequent disappearance of these events that was accompanied by a progressive reduction of amplitude, but a prolongation in time-course, of the evoked Ca^2+^ transients with time. The former observation was consistent with a reduction in store Ca^2+^ levels available for release by electrical stimulation consistent with a delayed reduction of SR Ca^2+^ and consistent with earlier studies that have actually used caffeine as a means of depleting these stores.^35^ Caffeine also abolished spatial heterogeneities in the Ca^2+^ transients consistent with actions on the CICR process.^21^,^30^–^32^,^38^ These findings parallel the more frequent spontaneous quantal Ca^2+^ release events (sparks) and Ca^2+^ waves in atrial cardiomyocytes from AF patients.^3^ Similarly, abnormal function in atrial Ca^2+^ release channels resulting in increased open probabilities and diastolic leak of Ca^2+^ relates to an increased triggered activity in heart failure.^39^

These findings were corroborated by observations from experiments using the complementary agents CPA and nifedipine. Cyclopiazonic acid is thought to inhibit Ca^2+^-ATPase activity and ultimately reduce SR Ca^2+^.^40^ In the present study, CPA pretreatment reduced the evoked Ca^2+^ signals without altering their kinetics or spatial heterogeneities and inhibited the diastolic Ca^2+^ events caused by the subsequent addition of caffeine. These findings similarly suggest a depletion by CPA of SR Ca^2+^ releasable by electrical stimulation that also results in a reduction in caffeine-induced diastolic events. Nifedipine is a known competitive dihydropyridine LTCC blocker in ventricular cells with a *K*_D_ of 40 nmol/L;^23^,^41^ therefore, nifedipine would be expected to diminish extracellular Ca^2+^ entry.^23^,^25^ Nifedipine produced immediate reductions in evoked Ca^2+^ transients while preserving their kinetics and spatial heterogeneity. Subsequent addition of caffeine similarly restored the amplitudes of the evoked Ca^2+^ transients, again without the expected induction of diastolic Ca^2+^ transients, consistent with CICR reduced by inhibited entry of extracellular Ca^2+^. These findings at the cellular level correlate with the presence or absence of atrial arrhythmogenicity in whole hearts. The presence or absence of spontaneous and provoked atrial arrhythmogenesis were examined under conditions of intrinsic and regular pacing from the right atrium and using programmed electrical stimulation, respectively. Control hearts showed no evidence of arrhythmogenicity, whether during intrinsic or regular pacing, and a low incidence of AT during programmed electrical stimulation, recapitulating previous clinical observations of brief periods of AT that follow the imposition of extra stimuli.^27^

In contrast, the addition of caffeine initially resulted in episodes of both AT and AF during regular pacing and an increased incidence of arrhythmogenic phenomena during PES in intact hearts, but this effect disappeared over the next 5min. These findings are directly comparable with the early appearance of diastolic Ca^2+^ release attributable to an enhanced CICR, but their subsequent disappearance is due to the consequent SR Ca^2+^ reduction. This would be consistent with the correlations between the observed abolition of atrial arrhythmogenicity in the whole hearts by CPA, which appeared to reduce myocyte SR Ca^2+^ even in the presence of caffeine. Finally, the effects of nifedipine in inhibiting caffeine-induced arrhythmogenesis were clearly correlated with its actions in reducing extracellular Ca^2+^ entry, which would similarly reduce CICR, albeit through a different mechanism.

The presence of caffeine continued to result in prolonged Ca^2+^ transient kinetics at the cellular level, as evidenced in our observations of increased FWHM values despite the reduced Ca^2+^ peaks. Nevertheless, such changes in FWHM indicate changes in the kinetics as opposed to the quantity of Ca^2+^ release. They confirm actions of caffeine in atrial cells not shared by the other agents tested. Thus, CPA and nifedipine alone, thought to act primarily on SR Ca^2+^ reuptake and voltage-dependent Ca^2+^ entry, respectively, produced no observable changes in FWHM. Thus, the results are consistent with the action of caffeine on CICR that has been established previously for ventricular cells.^28^ Finally, the actions of caffeine, CPA and nifedipine together on the heterogeneity of the observed Ca^2+^ signals also implicate changes in CICR: caffeine abolished such heterogeneities, whereas CPA and nifedipine did not.

Taken together, the findings from single cells and intact hearts suggest acute atrial arrhythmogenic phenomena that are dependent on a diastolic release of SR Ca^2+^, itself dependent upon a finite SR Ca^2+^ store and initiation of Ca^2+^ entry, both of which may then offer possible therapeutic targets in the clinical management of acute AF. We have demonstrated that caffeine produces early arrhythmogenesis in intact hearts. This correlates with the early appearance of diastolic Ca^2+^ transients in single cells, suggesting an immediate cause for this arrhythmogenesis. However, caffeine also produces a progressive decline in Ca^2+^ release, consistent with a depletion of cellular SR Ca^2+^ stores. It is then possible to demonstrate increased FWHM, not seen either when SR Ca^2+^ is depleted by CPA or in the presence of nifedipine. This is consistent with an action of caffeine on atrial CICR and possible roles of this CICR in atrial arrhythmogenesis. Therefore, the findings of the present study directly complement earlier studies that explored the effects of pharmacological changes of Ca^2+^ homeostasis.^28^,^36^
